# VARIETAL IDENTIFICATION IN HOUSEHOLD SURVEYS: RESULTS FROM THREE HOUSEHOLD-BASED METHODS AGAINST THE BENCHMARK OF DNA FINGERPRINTING IN SOUTHERN ETHIOPIA

**DOI:** 10.1017/S0014479718000030

**Published:** 2018-02-20

**Authors:** FRÉDÉRIC KOSMOWSKI, ABIYOT ARAGAW, ANDRZEJ KILIAN, ALEMAYEHU AMBEL, JOHN ILUKOR, BIRATU YIGEZU, JAMES STEVENSON

**Affiliations:** † CGIAR Standing Panel on Impact Assessment, Food and Agriculture Organization of the United Nations, Viale delle Terme di Caracalla, 00153 Roma RM, Italie; § International Potato Center (CIP), PO Box 10059, Addis Ababa, Ethiopia; ¶ Diversity Arrays Technology Pty. Ltd., Building 3, Level D, University of Canberra, Kirinari St. Bruce, ACT2617 (LPO Box 5067), Australia; †† The World Bank, 1818 H St. NW, Washington, DC 20433, USA; ‡‡ Central Statistical Agency of Ethiopia, Piassa, Addis Ababa, Ethiopia

## Abstract

Accurate crop varietal identification is the backbone of any high-quality assessment of outcomes and impacts. Sweetpotato (*Ipomoea batatas*) varieties have important nutritional differences, and there is a strong interest to identify nutritionally superior varieties for dissemination. In agricultural household surveys, such information is often collected based on the farmer’s self-report. In this article, we present the results of a data capture experiment on sweet potato varietal identification in southern Ethiopia. Three household-based methods of identifying varietal adoption are tested against the benchmark of DNA fingerprinting: (A) Elicitation from farmers with basic questions for the most widely planted variety; (B) Farmer elicitation on five sweet potato phenotypic attributes by showing a visual-aid protocol; and (C) Enumerator recording observations on five sweet potato phenotypic attributes using a visual-aid protocol and visiting the field. In total, 20% of farmers identified a variety as improved when in fact it was local and 19% identified a variety as local when it was in fact improved. The variety names given by farmers delivered inconsistent and inaccurate varietal identities. Visual-aid protocols employed in methods B and C were better than those in method A, but greatly underestimated the adoption estimates given by the DNA fingerprinting method. Our results suggest that estimating the adoption of improved varieties with methods based on farmer self-reports is questionable and point towards a wider use of DNA fingerprinting in adoption and impact assessments.

## INTRODUCTION

Developing countries rely on agricultural productivity for growth (World Bank, 2007), and achieving a Green Revolution in sub-Saharan Africa is a major objective of many development organizations. Indeed, the period of food growth production witnessed in the mid-1960s in several Asian countries has contributed to widespread poverty reduction, averted hunger for millions of people, and avoided the conversion of thousands of hectares of land into agricultural cultivation (Stevenson *et al.*, 2012). One essential activity of agricultural development is the breeding and dissemination of improved varieties. Crop germplasm improvement is thus a major activity of CGIAR centres and thousands of new varieties have been developed in different agro-ecological contexts to provide higher yield, better nutritional content or increase resistance to diseases or droughts. Accurate information on crop varieties is therefore crucial to study the extent of adoption by farmers and evaluate the performance of agricultural development programmes.

However, measuring and understanding the diffusion of improved crop varieties remains challenging. The challenge is more pronounced among poor smallholder farmers where records from official transactions are often missing. Various methodologies such as sales inquiries, expert opinion estimates and household survey questionnaires have been employed, each with their own inherent limitations (Abate *et al.*, 2017; Walker, 2015). For example, seed-sales inquiries require specific surveys, which may not fit into existing agricultural statistic systems. They are also more susceptible to recall bias. In addition, companies are often unwilling to share this information with the public. In a major effort to quantify the adoption of improved varieties in sub-Saharan Africa, the Diffusion and Impact of Improved Varieties in Africa (DIIVA) project has shed light on the convergence of expert opinion with household survey estimates (Walker, 2015). Conclusions point towards the fact that estimates based on expert opinion are likely to overemphasize the uptake of specific varieties, while household surveys are likely to understate their importance. The study concludes that ‘probably neither surveys nor expert panels can do a good job in delivering accurate estimates of cultivar-specific adoption’ (Walker, 2015). Assessing the extent of measurement errors is, however, impossible in the absence of an objective benchmark. Since 2010, the technology of DNA fingerprinting has become increasingly affordable, and costs per sample are projected to continue to decrease in the coming decade. The emergence of DNA fingerprinting as a survey instrument provides the opportunity to conduct a survey validation exercise and assess the accuracy of existing methods for collecting crop varietal identification (Maredia *et al.*, 2016; Rabbi *et al.*, 2015). However, the available evidence does not provide information on relevant questions. For example, does this survey validation exercise matter for all crops? Or is it different for different crops? How are different household survey based approaches performing against the DNA fingerprinting benchmark?

In this study, we focused on sweet potato varietal identification. While the Green Revolution was mainly based on the diffusion of crop genetic improvement for the three main staple cereals (maize, rice and wheat), sub-Saharan Africa exhibits a high diversity of crops, which are of similar importance for food security (Pingali, 2012). Among them, sweet potato has encountered widespread interest since the 1980s. Sweet potato is a co-staple crop in East Africa’s mid-elevation farming areas. In Ethiopia, the number of sweet potato producers has increased recently and the crop is now considered as a major food crop, with 1.6 million producers (Central Statistical Agency, 2012). It is mainly used for household consumption (82%), with only a small portion of the crop being sold (12%). Sweet potato seed system is almost entirely informal, with only occasional formal distributions of new varieties by agricultural research centres or non-governmental organizations (Namanda *et al.*, 2011). The crop is generally propagated from farmer to farmer by vine cuttings obtained from mature crops.

Sweet potato offers several advantages. The crop requires low levels of inputs, can grow on degraded soils and is easily propagated from vines. Sweet potato is often regarded as a food security crop, having a flexible growing season over a 3- to 10-month period. The crop is also good to cope with slack season because it is possible to harvest sweet potato before the harvest season for other crops, at times when food shortages are common. Finally, sweet potato is a candidate of choice for biofortification – the breeding of micronutrients into crops to control vitamin A, iron and zinc deficiencies (Bouis *et al.*, 2011).

Indeed, different varieties of sweet potatoes have different nutritional value. Orange-fleshed sweet potato varieties have high beta-carotene content and represent a promising and cost-effective way to combat micronutrient deficiencies, which are prevalent through the developing world. There is mounting evidence that the introduction of orange-fleshed sweet potato can increase vitamin A intakes among children and women (Hotz *et al.*, 2012) and reduce children’s diarrhoea prevalence and duration (Jones and de Brauw, 2015). With the objective of spreading an ‘orange revolution’, several projects have been implemented to promote and disseminate orange-fleshed varieties (HarvestPlus, 2012; Miethbauer *et al.*, 2015). Therefore, varietal information is important to accurately measure the health and nutrition implications of sweet potato diffusion.

In this study, we test the effectiveness of three household-based survey methods of identifying varietal adoption against the benchmark of DNA fingerprinting of sweet potato leaf samples. These are: (A) Elicitation from farmers with basic questions for the most widely planted variety; (B) Farmer elicitation on five sweet potato phenotypic attributes using a visual-aid protocol; and (C) Enumerator recording observations on five sweet potato phenotypic attributes from the visual-aid protocol by visiting the field.

## MATERIALS AND METHODS

### Sweet potato improved varieties released in Ethiopia

In Ethiopia, the term ‘improved variety’ is used to designate a variety that has been tested by breeders and evaluated for its superiority over existing (traditional or local) varieties (Ethiopian Ministry of Agriculture, 2013). The list of improved sweet potato varieties released in Ethiopia is provided in Table 1. Since 1990, a total of 25 improved sweet potato varieties have been released. Breeding and germplasm maintenance activities have been concentrated in the Southern Nations, Nationalities and Peoples’ Region (SNNPRS) and Oromia (Miethbauer *et al.*, 2015). Five orange-fleshed varieties have been released and promoted for their higher nutritional content: Koka-12, Guntutie, Kero, Kulfo and Tulla.

**Table 1:** Sweet potato improved varieties released by the national agricultural research system of Ethiopia, 1990–2013.

Variety	Year of release	Breeder
Tola	2012	Bako ARC
Ma’e	2010	Werer ARC
Jari	2008	Sirinka ARC
Birtukanie	2008	Sirinka ARC
Berkume	2007	Haramaya University
Adu	2007	Haramaya University
Balo	2006	Baco ARC
Ordollo	2005	Awassa ARC
Kero (OFV)	2005	Awassa ARC
Tulla (OFV)	2005	Awassa ARC
Kulfo (OFV)	2005	Awassa ARC
Dimitu	2005	Bako ARC
Temesgen	2004	Awassa ARC
Beletech	2004	Awassa ARC
Belela	2002	Awassa ARC
Awassa-83	1997	Awassa ARC
Dubo	1997	Awassa ARC
Falaha	1997	Awassa ARC
Kudadie	1997	Awassa ARC
Damota	1997	Adet ARC
Bareda	1997	Awassa ARC
Guntutie (OFV)	1997	Awassa ARC
Ogan-Sagan	unknown	Ministry of Agriculture
Koka-12 (OFV)	1987	Awassa ARC
Koka-6	1987	Awassa ARC

Source: Ethiopian Ministry of Agriculture, 2013. ARC = Agricultural research centre. OFV = Orange-fleshed variety.

Crop descriptors follow a standard codification and are regarded as a universally understood language for germplasm data. The International Board for Plant Genetic Resources recommends a list of 26 descriptors related to the plant morphology, storage root and inflorescence (CIP, AVRDC, IBPGR, 1991). However, several descriptors can be tricky to assess for non-specialists. In particular, many descriptors are recorded as an average value of measurement (for instance, length or size) or an average expression of the character. In contrast with most crops, sweet potato varieties exhibit a diversity of colours on different parts of the plant as well as heterogeneity of leaf shapes. This makes the crop particularly interesting to test a visual-aid survey protocol based on distinctive phenotypic attributes. To this purpose, available documents on the descriptors of sweet potato improved varieties were reviewed, and interviews with specialists were conducted. Based on discussions with breeders, observation of plots and pre-testing of different protocols, we identified five phenotypic attributes that are relevant for sweet potato varietal identification and are more likely to be perceived by interviewees and enumerators (Supplementary Table S1, available online at https://doi.org/10.1017/S0014479718000030.). Indeed, visual-aid protocols offer advantages over the existing methods of data collection. Pictures have the potential to overcome language and translation barriers, which could be a huge advantage on data quality. Earlier in the project, a visual aid protocol had been included in the Ethiopian Socioeconomic Survey (ESS) implemented in 2015/16 by the Ethiopian Central Statistical Agency and the World Bank on a nationally representative sample of 3800 rural households. In this study, visual-aid protocols, collecting information on the sweet potato variety skin colour, flesh colour, dominant type leaf shape, vein colour and vine colour, were used as a survey instrument (Figure S1).

### Varietal identification by phenotypic attributes

**Figure 1:**
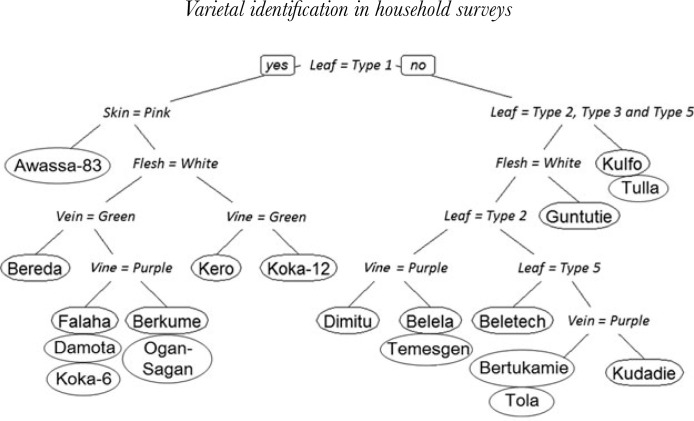
Varietal identification of sweet potato improved varieties using classification tree analysis. If the answer is yes, the left branch follows; if no, the right branch follows.

Recursive partitioning methods and classification trees (Breiman *et al.*, 1984) provide a potential way to uniquely identify improved varieties on the basis of their descriptors. In our case, the response variable is a 19-level categorical variable of sweet potato improved varieties, while the five phenotypic attributes are used as explanatory variables. The analysis, which generates a set of decision rules and predict varieties, is described in Therneau *et al*. (2015). The first step is identifying the single variable that best splits the data into two groups. The data are separated, and then this process is applied separately to each sub-group, and so on, recursively until the subgroups either reach a minimum size or until no improvement can be made. The second step of the procedure consists of using cross-validation to trim back the full classification tree. Results of the classification tree analysis are presented in Figure 1. Overall, the algorithm identified 13 different paths out of the 19 varieties for which germplasm were collected and included in the reference library used for DNA fingerprinting. Eight sweet potato improved varieties are uniquely identified by the classification tree, while the remaining 11 share common phenotypic traits with other varieties.

### Data collection

Field data were collected in January 2015 in Wolayita zone, a major sweet potato producing area in Ethiopia. Compared to the national average of 7.600 kg/ha, sweet potato yields in the Wolayita area averaged at 10.700 kg/ha in the 2011/12 agricultural season (Central Statistical Agency, 2012). The survey was implemented in five different communities (kebelles): Buge, Ade Koyisha, Gacheno, Waja Kero and Ofa Sere (Figure S2) using snowball sampling. In each community, an initial group of sweet potato growers was identified with the help of local authorities. These farmers were then asked to provide information to locate other sweet potato growers in the area. Although snowball sampling may introduce a bias in our sample, we are more interested by varietal diversity than by representativeness. Oral consent was granted from all participants and the data were analysed anonymously. Tablets equipped with the Open Data Kit application were used. The survey questionnaire included three modules. Module-1 captured information on the most widely grown variety, followed by basic questions on this variety. The variety name given by the interviewee was repeated in each question throughout the questionnaire. Farmers were asked to report whether the sweet potato variety grown is a local or improved variety – referring as ‘yakabababe zer’ for a local variety and ‘mirit zer’ for an improved one – and whether the variety was introduced by the government. In Module-2, the interviewees were asked about phenotypic attributes of the main variety they are growing, using the visual-aid protocol. The visual aid was presented to interviewees to identify the variety attributes while the plot was not within view. Then, the enumerator was accompanied by the farmer to the plot to answer Module-3, and the same five attributes were recorded by the enumerator. The plots were georeferenced and leaf tissues from 259 fields were collected with a unique ID and placed into a plastic bag with silica gel. At the end of the interview, farmers were asked to help in identifying other sweet potato growers around the area.

### DNA extraction and genotyping by sequencing

Samples collected from the farmer’s fields were freeze dried and processed. Collected samples as well as the genotypes included in the reference library were extracted according to the CetylTrimethylAmmonium Bromide (CTAB) method (Kilian *et al.*, 2012). The quality and concentration of the extracted DNA were checked on agarose (1%) against a known concentration of Lamda DNA (10, 30 and 50 ng). To establish the library, we included 19 improved materials collected from the agricultural research centres of Awassa, Adami Tulu and Baco. Six improved materials could not be included in the reference library because they were either not maintained anymore on research stations (Ordollo, Dubo, Adu and Balo) or were unlikely to be found in the variety collection area (Ma’e and Jari). Additionally, 1004 lines from the CIP genebank accessions in sub-Saharan Africa were included in the reference library. In practice, however, only a limited number of samples could be matched with the CIP accessions.

For genotyping by sequencing, a combination of a diversity arrays technology (DArT) complexity reduction methods and next generation sequencing platforms was used (Kilian *et al.*, 2012; Raman *et al.*, 2014). Following the PstI–MseI method, sweet potato DNA samples were processed in digestion/ligation reactions principally as per Kilian *et al*. (2012) but replacing a single PstI-compatible adaptor with two different adaptors corresponding to two different restriction enzyme (RE) overhangs. The PstI-compatible adapter was designed to include Illumina flowcell attachment sequence, sequencing primer sequence and ‘staggered’, varying length barcode region, similar to the sequence reported by Elshire *et al*. (2011). Reverse adapter contained flowcell attachment region and MseI-compatible overhang sequence. Only ‘mixed fragments’ (PstI–MseI) were effectively amplified in 30 rounds of polymerase chain reaction (PCR). After PCR, equimolar amounts of amplification products from each sample of the 96-well microtiter plate were bulked and applied to cBot (Illumina) bridge PCR followed by sequencing on Illumina HiSeq 2000. The sequencing (single read) was run for 77 cycles.

Sequences generated from each lane were processed using proprietary DArT analytical pipelines (Sánchez-Sevilla *et al.*, 2015; Sansaloni *et al.*, 2011). In the primary pipeline, the fastq files were first processed to filter away poor quality sequences, applying more stringent selection criteria to the barcode region compared to the rest of the sequence. In that way, the assignments of the sequences to specific samples carried in the ‘barcode split’ step were very reliable. Approximately 1.4 million sequences per barcode/sample were identified and used in marker calling. Finally, identical sequences were collapsed into ‘fastqcoll files’. The fastqcoll files were ‘groomed’ using DArT PL’s proprietary algorithm, which corrects low quality base from singleton tag into a correct base using collapsed tags with multiple members as a template. The ‘groomed’ fastqcoll files were used in the secondary pipeline for DArT PL’s proprietary single nucleotide polymorphism (SNP) markers and SilicoDArT (presence/absence of restriction fragments in representation) calling algorithms (DArTsoft14). For SNP calling all tags from all libraries included in the DArTsoft14 analysis are clustered using DArT PL’s C++ algorithm at the threshold distance of 3, followed by parsing of the clusters into separate SNP loci using a range of technical parameters, especially the balance of read counts for the allelic pairs. In addition, multiple samples were processed from DNA to allelic calls as technical replicates and scoring consistency were used as the main selection criteria for high-quality/low error rate markers. From the 259 leaf tissues collected, a total of 231 samples were DNA fingerprinted and matched with the reference library (File S1).

To determine the accuracy of the three household-based survey methods, the DNA fingerprinting method is considered as the benchmark. Information from the DNA fingerprinted samples was matched with the estimates provided by each method of data collection. Bivariate analysis is then used to assess the accuracy of data on variety type (improved *vs* traditional), variety name and variety phenotypic attributes.

## RESULTS

DNA analysis identified 63% of samples from farmers’ fields as improved varieties. Five improved varieties were identified in the surveyed area: Awassa-83, Berkume, Kudadie, Ogan-Sogan and Kulfo/Tulla. The most common improved types were Kulfo/Tulla (22%), Awassa-83 (20%) and Ogan-Sagan (12%). The remaining improved varieties were identified on a few number of samples only.

### Genetic diversity and population structure

**Figure 2:**
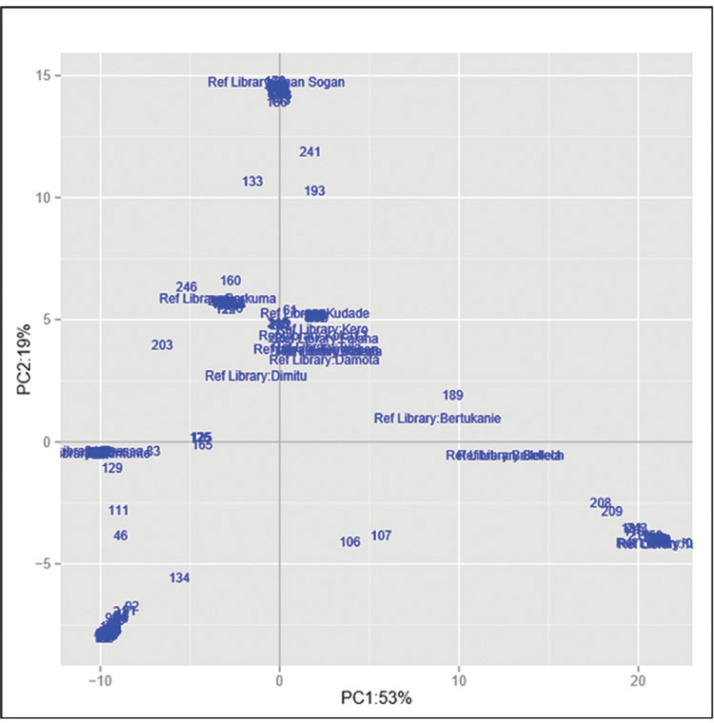
Genetic distance-based plot of the first two axes of the principal component analysis (PCA) based on the reference library and the collected samples.

The set of samples was characterized using 17,220 genome-wide DArT markers. The Neighbor-Join Phylogram obtained from DArT markers is shown in Figure S3. Furthermore, the genetic divergence among samples was analysed using principal coordinate analysis (Figure 2). The first axis (*x*) expresses 53% of the total variation and the second axis 19%. It is apparent that most varieties from the reference library, displayed around the plot centre, showed relatively similar genetic profiles. Ogan-Sogan samples, likely the oldest released variety, were located along the top of the second axis, showing high genetic distance with other varieties. By contrast, Berkume and Awassa-83 varieties were located in the left quadrant, with Awassa-83 having the highest genetic distance. Tulla and Kulfo, two orange-fleshed varieties released in 2005, showed a similar genetic profile (bottom of the right quadrant). Several local varieties did not match the reference library and were located at the bottom of the left quadrant.

### Estimates of methods A, B and C against DNA fingerprinting

Table 2 summarizes the accuracy of estimates of the adoption of improved varieties by each method of data collection. The accuracy of data derived from the three methods is evaluated against the benchmark of varietal identification established through DNA fingerprinting.

**Table 2:** Summary results of improved varieties adoption estimates established through DNA fingerprinting and derived from the three methods.

	Method A^*^	Method B	Method C
% True positive (improved when improved)	43	47	50
% True negative (local when local)	16	4	6
% False positive (Type I Error: improved when local)	20	33	31
% False negative (Type II Error: local when improved)	19	16	13

* 2% of respondents did not know whether the variety is improved or local.

The first result is that all methods appear less accurate than the DNA fingerprinting benchmark. Method A suggests a less accurate identification of improved varieties by farmer’s elicitation (Is this variety a traditional or improved variety?): In total, 20% of farmers identified a variety as improved when in fact it was local, and 19% identified a variety as local when it was in fact improved.

As an alternative way of identifying improved varieties, the survey asked: ‘Has this variety been introduced by the government?’. Results demonstrate that this question was even less accurate, with less than half of improved varieties (46%) identified as such by this alternative question. Based on improved varieties phenotypic attributes, methods B and C, respectively, identified 47 and 50% of improved varieties correctly, thus representing a slight improvement in accuracy over the existing method of collection, method A. These two methods, however, delivered a higher number of false negative results.

### Method A: Interviewee’s self-report without visual aid

**Figure 3:**
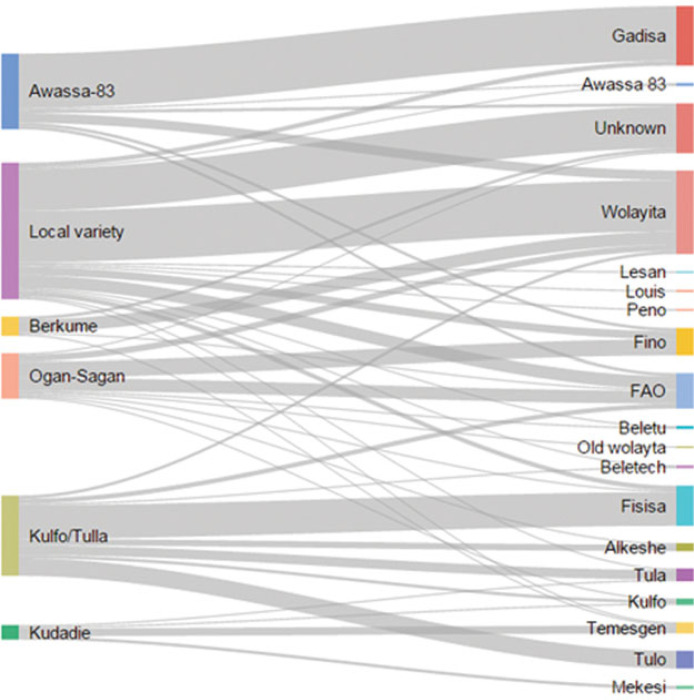
Sankey diagram capturing the relationship between sweet potato varieties identified through DNA fingerprinting (left) and sweet potato variety names given by farmers (right). The bars indicate percentage of total varieties, while lines describe the relationship.

Three quarter of farmers grew only one variety. Over 18 different names were given by farmers to describe the varieties of sweet potato they planted. Most cited varieties were Wolayita, Gadissa, Fisisa and FAO. One-tenth of interviewees could not name the variety they grew. As shown in Figure 3, the variety names given by farmers mapped inconsistently to improved varieties: only 6% of improved varieties were correctly identified by name. It is notable that some varieties (Awassa-83 and Kulfo/Tulla) are largely linked to a single name. We also note that the common response to a local variety name that the respondents provided were ‘Wolayita’ (the name of the area) and ‘unknown’. However, there is no consistent pattern regarding the extent and direction of the error – whether adoption of a specific variety is over- or under-estimated. While one could expect a lower misclassification rate for varieties that have very distinct phenotypic traits (orange flesh colour, pink skin colour or specific leaf type), this does not appear to be the case. Ogan-Sagan and Berkume, two varieties with very common traits, are reported by farmers under nine and four different names, respectively. On the other side, orange-fleshed varieties are misclassified with six other names, while Awassa-83 (pink flesh) appears under five other names. These results would suggest that in informal seed systems, as in the case of sweet potato in southern Ethiopia, two issues arise when attempting to identify improved varieties by name. First, farmers may refer to a specific improved variety by giving it a different name. The name of the agricultural officer who promoted the variety is often accepted as the variety’s name and this case is encountered for Awassa-83, largely referred as ‘Gadissa’, and Kulfo/Tulla, referred as ‘Fisisa’. Second, it is difficult to rule out misspelling, which may result in several misclassification cases. For example, the names ‘FAO’ and ‘Fino’ or ‘Tula’ and ‘Tulo’, which sound similar, would typically point us to be suspicious of the presence of widespread measurement error.

### Methods B (interviewee with visual-aid) and C (enumerator observation)

Although method B represents an improvement in accuracy over farmer’s elicitations (where only 6% of improved varieties were correctly identified by name), there is still a large amount of measurement errors. Farmer’s answers on phenotypic characteristics rarely match the correct varieties and information provided by the visual-aid protocol failed to uniquely identify improved varieties (Table 3). In all cases except Kulfo/Tulla, having an enumerator visiting the field only provided a small improvement over asking the interviewee the question directly, and method C also provided results that are way below the DNA fingerprinting benchmark. Another important observation in Table 3 is that varieties with colourful attributes such as Kulfo/Tulla were more easily identified: out of 50 samples, 38 orange-fleshed varieties were correctly identified by both methods B and C.

**Table 3:** Varietal identification of sweet potato improved varieties established through DNA fingerprinting and derived from the three methods (*n* = 146).

	Method A	Method B	Method C
Awassa-83 (*n* = 47)			
Correct	1	15	25
False positive	46	32	22
False negative	1	3	7
Berkume (*n* = 12)			
Correct	0	2	5
False positive	12	10	7
False negative	0	4	6
Kudadie (*n* = 9)			
Correct	0	0	0
False positive	9	9	9
False negative	0	5	8
Kulfo/Tulla (*n* = 50)			
Correct	8	38	38
False positive	42	12	12
False negative	3	5	6
Ogan-Sagan (*n* = 28)			
Correct	0	7	9
False positive	28	21	19
False negative	0	2	9

**Figure 4:**
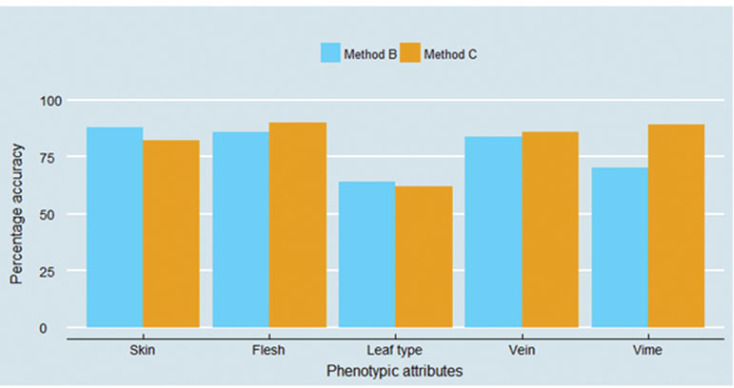
Accuracy of data collected on five sweet potato phenotypic attributes.

It is important to understand which phenotypic attributes were accurately identified by both methods, and which ones were not. Figure 4 explores this question, and delivers three important messages. First, skin, flesh and vein colours were perceived by interviewees as well as enumerators more than 80% of the cases. Second, of all phenotypic attributes, data collected on leaf types were found to be the most inaccurate. Among the different types, the hand-shaped leaf type, typical for the orange-fleshed varieties Kulfo and Tulla, was the only one to be easily identified by interviewees (84% accuracy) and enumerators (86% accuracy). Other leaf types demonstrate poor identification: only half of hearth-shaped leaf type were correctly identified by both methods of data collection, and the leaf type 4, typical of the Kudadie variety, was accurately identified by 56% of interviewees and only one-third of enumerators. However, as the five improved varieties identified through DNA fingerprinting only have three different types of leaves, we were not able to explore all sweet potato leaf types. Finally, with the exception of the vine colour, having the enumerator visiting the field provided only a slightly better (flesh and vein) or even a slightly lower (skin and leaf type) accuracy over the farmer’s response.

## DISCUSSION

The objective of this study was to compare different methods of data collection for sweet potato varietal identification. The gold standard represented by DNA fingerprinting validation is compared to other low-cost, easy to implement methods. Crop germplasm improvement is a major activity of agricultural research centres throughout the world and varietal identification is central to assess its contribution and impact. In addition, different varieties of sweet potatoes have different nutritional value and there is a strong interested among development agents in assessing the extent of biofortified sweet potato varieties adoption. All methods were found to be less accurate than the DNA fingerprinting benchmark. Data quality may suffer since information from these methods proves to be unreliable. Regarding sweet potato varietal identification, a wider use of DNA fingerprinting seems unavoidable.

Implemented throughout sub-Saharan Africa, household surveys are the most common source of data for modern crop varietal adoption (Abate *et al.*, 2017; Afolami *et al.*, 2015; Asfaw *et al.*, 2012). The surveys typically ask the most knowledgeable person in the sampled farm household. However, the results presented in this study show that farmers were not able to identify improved varieties (Table 2). Moreover, farmer’s identification of improved varieties by name only delivered inconsistent varietal identification. These results provide another piece in the puzzle of our view that current methods relying on farmer’s elicitation deliver biased estimates. Similar to our findings, recent studies with various crops in different sub-Saharan countries have demonstrated that, when compared to DNA fingerprinting, estimates of crop varietal adoption based on variety names and variety types (local *vs* improved) frequently diverge, often with large misclassification rates (Labeyrie *et al.*, 2014; Maredia *et al.*, 2016; Naino Jika *et al.*, 2017; Wossen *et al.*, 2017).

The fact that most, if not all, agricultural surveys rely on farmer’s elicitation raises concerns about the accuracy of the data collected by the traditional approaches. These results may highlight the importance of social factors and plant crop exchanges between farmers. It is understandable that a variety adopted by a farmer decades ago would be described as local, while it is in fact an improved variety that has been introduced as a result of a process of publically funded agricultural research. The informal nature of sweet potato seed system makes it even harder for farmers to assess sweet potato variety types. Whether household surveys under- or over-estimate adoption is context- and crop-specific and staple crops such as maize, wheat or barley may be more accurately identified by farmers.

This article contributes to the literature by introducing an innovative and reproducible method to track orange-fleshed sweet potato improved varieties. Visual-aid varietal identification protocols are low-cost and have the potential to fit into many existing agricultural surveys (Figure S1). In addition, using pictures overcomes language barriers – an important constraint throughout sub-Saharan Africa. Since the visual-aid protocols employed in methods B and C delivered adoption estimates that were far below those given by the DNA fingerprinting method, the question whether visual-aid protocols do represent a useful tool for tracking improved varieties deserves to be asked. Our results also indicate that visual-aid protocols based on colours may perform better than those relying on shapes and forms of plants attributes (Figure 4). The development of visual-aid protocols in other contexts should be encouraged: this method is certainly helpful in identifying varieties that have very distinctive phenotypic attributes, as it is the case with orange-fleshed sweet potatoes, and can offer low-cost improvements in data quality over traditional survey questions.

Our study is not without limitations. First, it is clear that more evidence is needed in different contexts, and for a higher variety of crops. It should be noted that methods A, B or C could work for other crops and other seed systems, so further experimentation should not be ruled out. Our survey data do not allow us to explore the relationship between self-report errors by farmers and observable characteristics of the farmer. While one could hypothesize that, for example, better educated farmers would be more able to provide accurate answers, arguably the informality in the seed system is the more binding constraint to more accurate survey-based identification.

As a viable tool to obtain accurate estimates of modern variety adoption, the use of DNA fingerprinting should be encouraged in future studies. Its implementation in large-scale household surveys in sub-Saharan Africa represents a substantial challenge but one that is worthy of significant future research efforts. Without the combination of accurate varietal identification and comprehensive socioeconomic and agricultural data for the same farms, assessing the adoption and impact of improved varieties (on productivity, and further to income effects for farmers) remains a formidable challenge. More and more countries are acquiring the technical capacities to extract DNA from field samples and to carry out genotyping. In addition, the costs of DNA fingerprinting are declining and will continue to do so in the coming decade. In the meantime, more evidence is needed to assess whether DNA fingerprinting should be used as a complementary or an essential part of crop varietal identification.

## SUPPLEMENTARY MATERIAL

To view supplementary material for this article, please visit https://doi.org/10.1017/S0014479718000030
