# Supplementary material for: VARIETAL IDENTIFICATION IN HOUSEHOLD SURVEYS: RESULTS FROM THREE HOUSEHOLD-BASED METHODS AGAINST THE BENCHMARK OF DNA FINGERPRINTING IN SOUTHERN ETHIOPIA
Source: Exp Agric. 2018 Feb 20;55(3):371–85. doi: 10.1017/S0014479718000030 (PMC7680950; doi:10.1017/S0014479718000030)
Supplement: Supplementary file 1 [file EA-55-03-371-s001.pdf]

1. When *[VARIETY NAME]* is cut after harvest, what is the <u>color of the flesh</u>? [__]

**1 = White**	**2 = Orange**
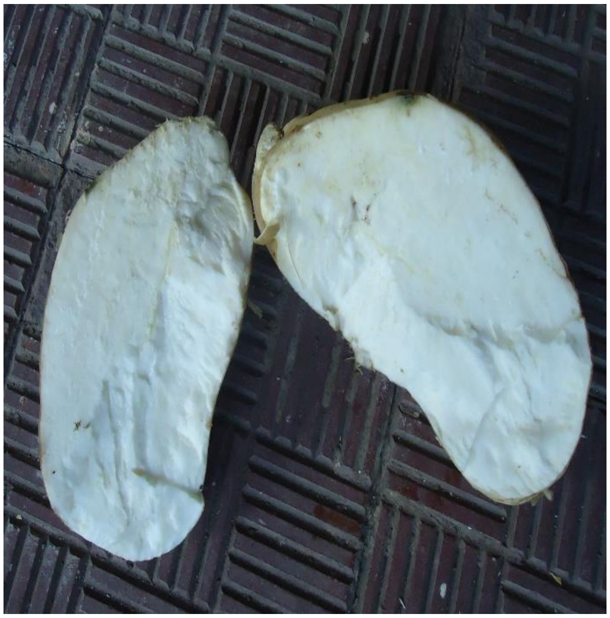	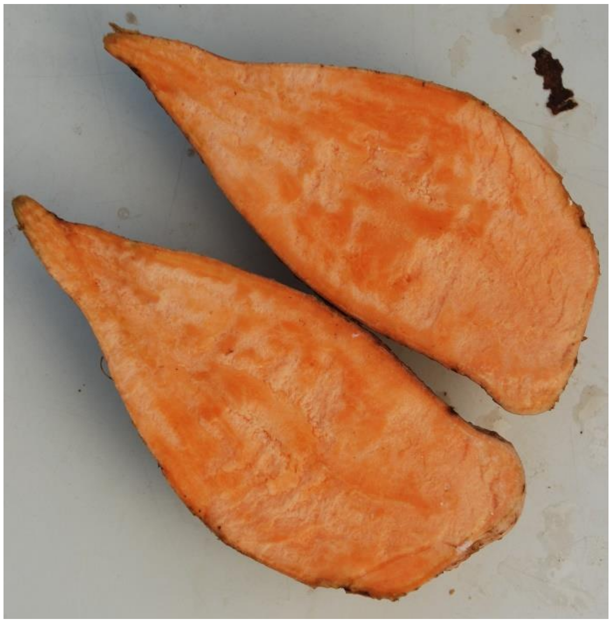

2. After *[VARIETY NAME]* has been harvested, what is the <u>color of the skin</u>? [__]

**1 = White**	**2 = Pink/Red**
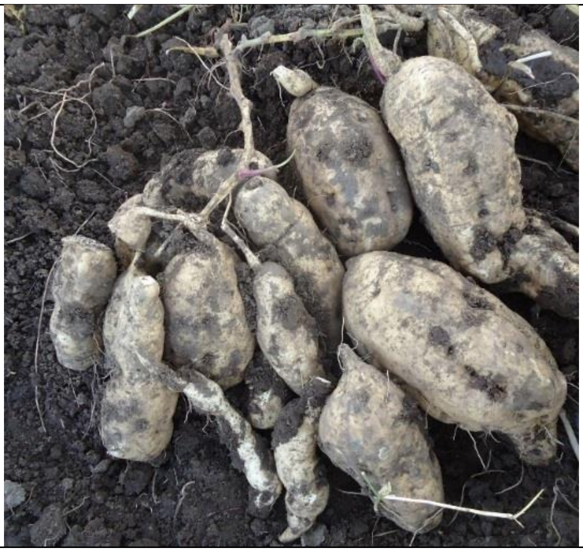	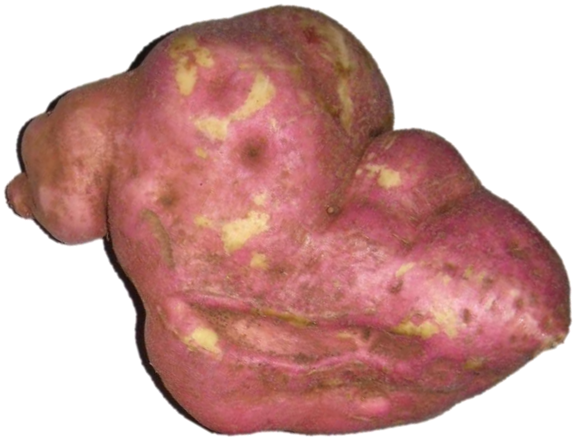

3. What is the dominant <u>shape of leaves</u> for *[VARIETY NAME]?* [__]

**1 = Hearth-shaped/triangular**	**2= with 3 nodes**
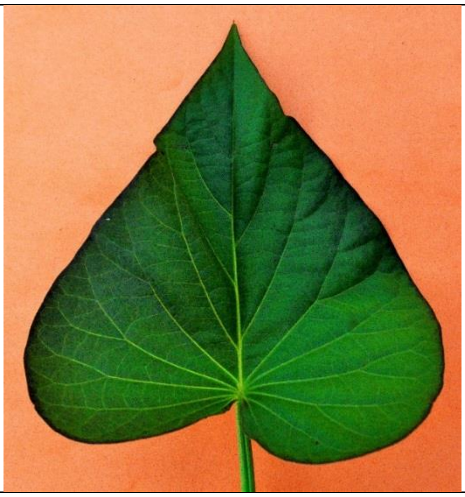	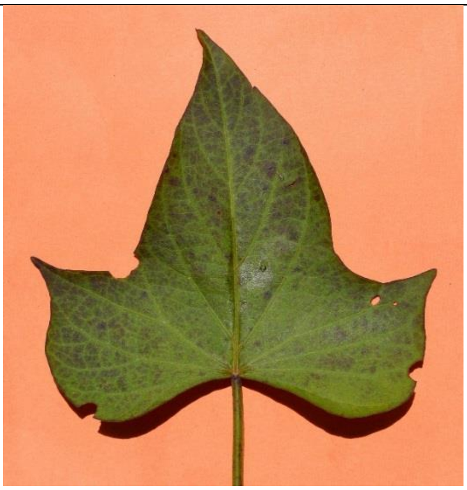
**3 = Hand-shaped – 5 fingers (narrow)**	**4 = with 5 fingers (1 major and 4 minors)**
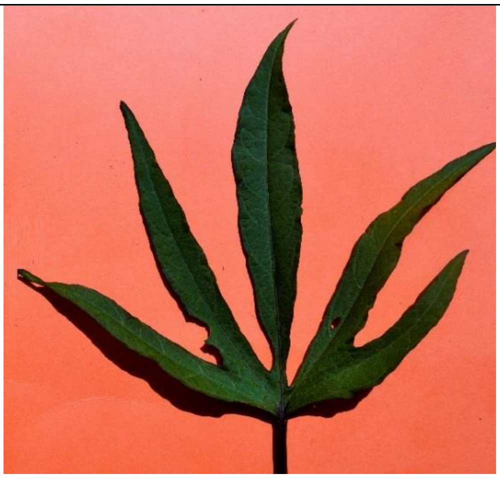	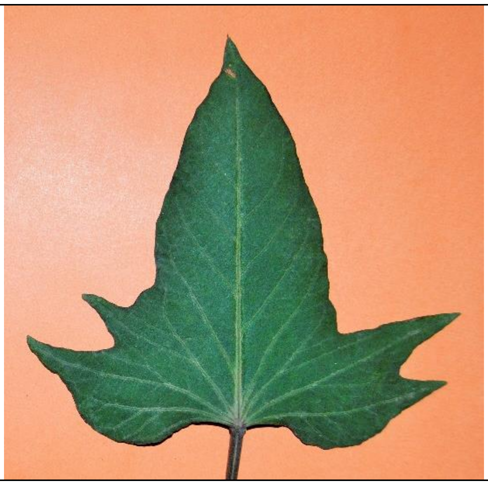
**5 = Hearth-shaped with teeth**	**6 - with 5 fingers**
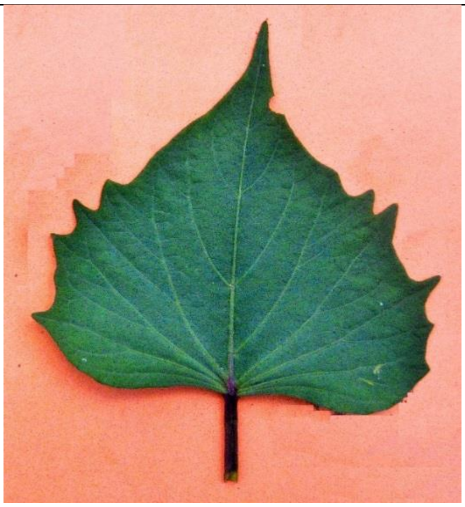	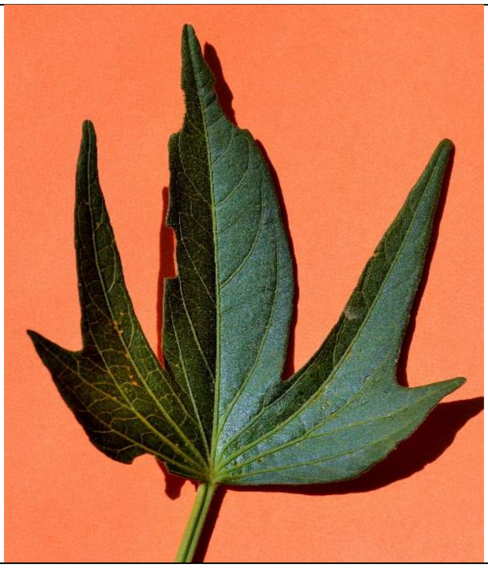

4. What is the <u>color of the vime</u> for *[VARIETY NAME]*? [__]

1 = Green	2 = Purple
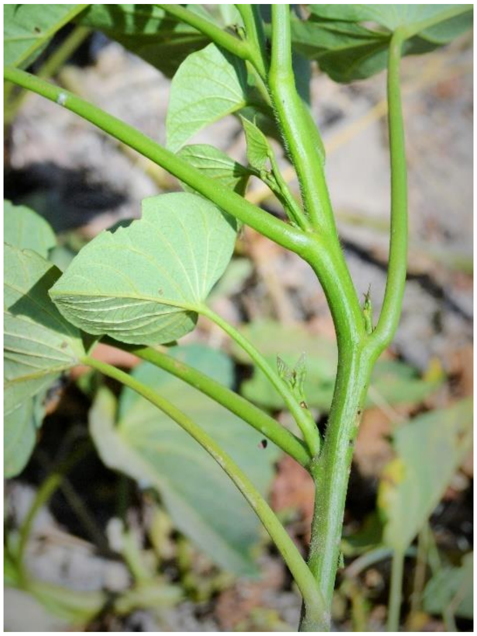	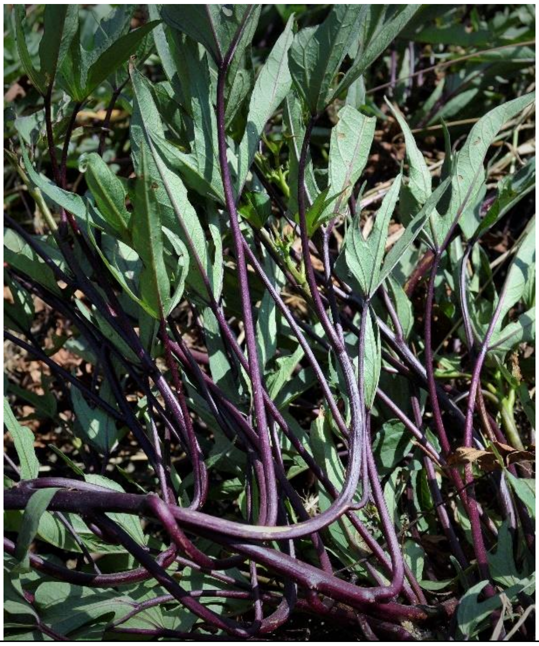

5. What is the <u>color at the back of leaves</u> for *[VARIETY NAME]*? [__]

1 = Green	2 = Purple
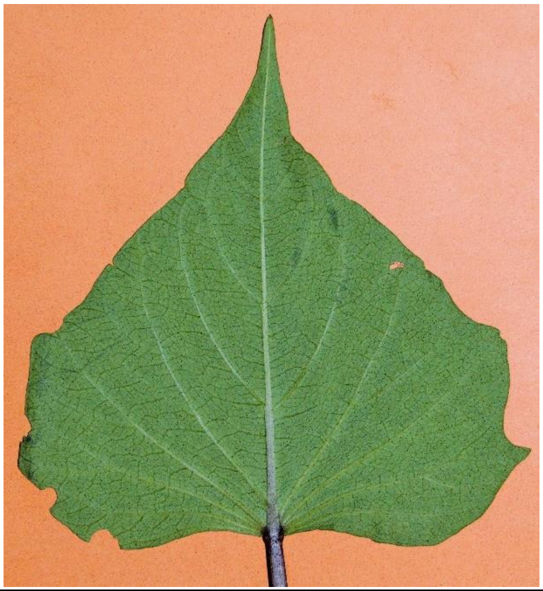	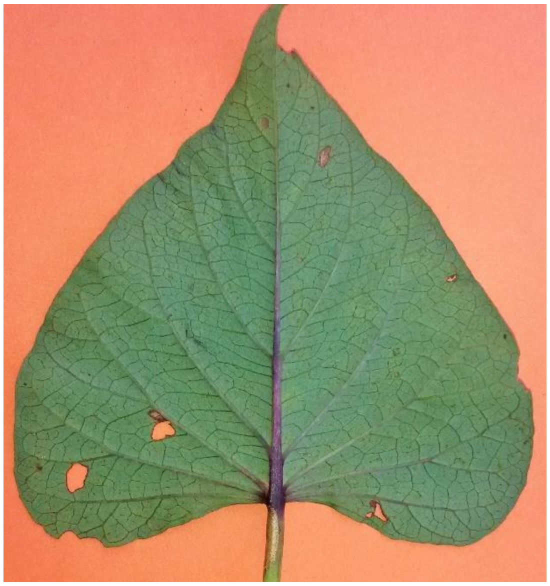
