# Supplementary figures and images for: VARIETAL IDENTIFICATION IN HOUSEHOLD SURVEYS: RESULTS FROM THREE HOUSEHOLD-BASED METHODS AGAINST THE BENCHMARK OF DNA FINGERPRINTING IN SOUTHERN ETHIOPIA
Source: Exp Agric. 2018 Feb 20;55(3):371–85. doi: 10.1017/S0014479718000030 (PMC7680950; doi:10.1017/S0014479718000030)

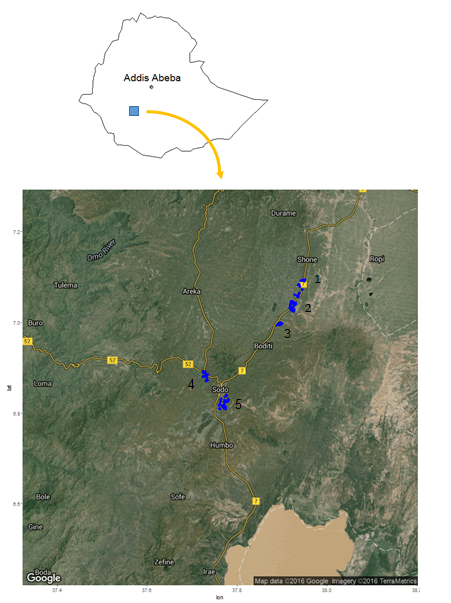

Supplement: Supplementary file 2 [file EA-55-03-371-s002.tif]

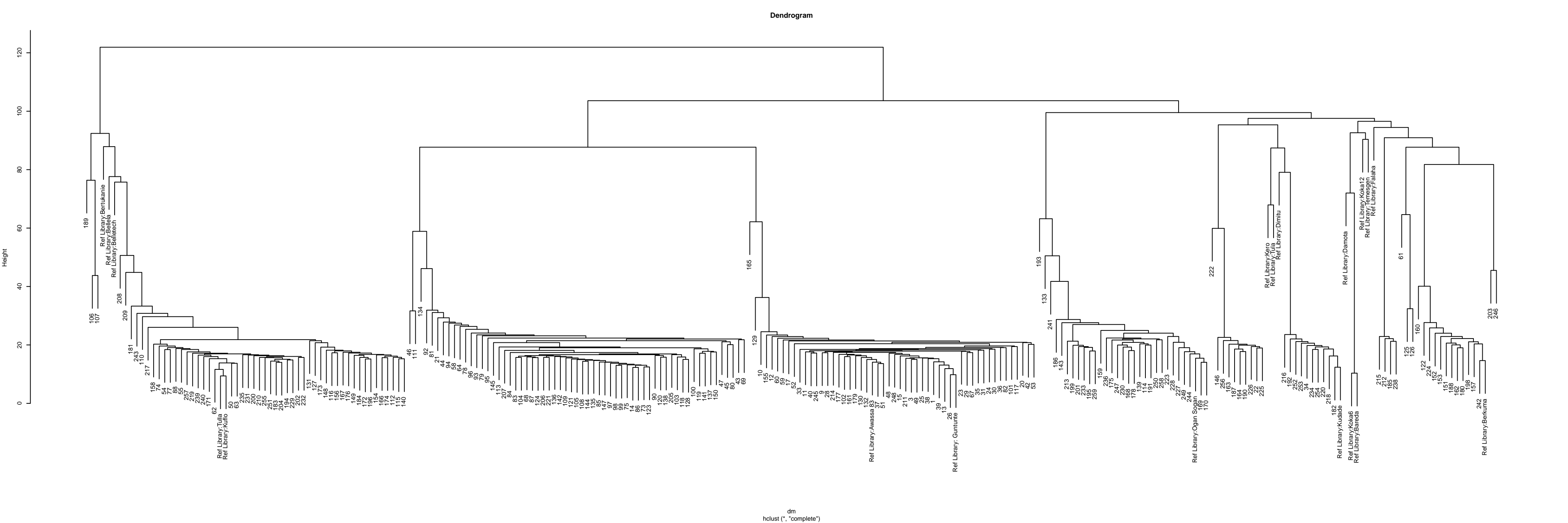

Supplement: Supplementary file 3 [file EA-55-03-371-s003.pdf]
