# Supplementary material for: VARIETAL IDENTIFICATION IN HOUSEHOLD SURVEYS: RESULTS FROM THREE HOUSEHOLD-BASED METHODS AGAINST THE BENCHMARK OF DNA FINGERPRINTING IN SOUTHERN ETHIOPIA
Source: Exp Agric. 2018 Feb 20;55(3):371–85. doi: 10.1017/S0014479718000030 (PMC7680950; doi:10.1017/S0014479718000030)
Supplement: Supplementary file 5 [file EA-55-03-371-s005.docx]

**Table S1.** Phenotypic attributes of sweet potato improved varieties released in Ethiopia.

| Variety | Flesh color | Skin color | Leaf type | Vine color | Leaf vein color |
| --- | --- | --- | --- | --- | --- |
| Awassa-83 | White | Pink | Type1 | Green | Green |
| Bareda | White | White | Type1 | Green | Green |
| Berkume | White | White | Type1 | Green | Purple |
| Damota | White | White | Type1 | Green | Green |
| Falaha | White | White | Type1 | Purple | Purple |
| Kero | Orange | White | Type1 | Green | Purple |
| Koka-12 | Orange | White | Type1 | Purple | Purple |
| Koka-6 | White | White | Type1 | Green | Green |
| Ogan Sagan | White | White | Type1 | Green | Purple |
| Belela | White | White | Type2 | Green | Green |
| Dimitu | White | Pink | Type2 | Purple | Green |
| Dubo | White | White | Type2 | Green | Green |
| Temesgen | White | White | Type2 | Green | Green |
| Kulfo | Orange | White | Type3 | Purple | Purple |
| Tulla | Orange | White | Type3 | Purple | Purple |
| Birtukanie | White | White | Type4 | Green | Purple |
| Guntutie | Orange | White | Type4 | Green | Green |
| Kudadie | White | White | Type4 | Green | Green |
| Tola | White | White | Type4 | Green | Purple |
| Beletech | White | White | Type5 | Purple | Green |
